# Association between sociodemographic characteristics and knowledge and practice of COVID-19 measures among households in Mombasa and Kilifi County, Kenya

**DOI:** 10.1093/inthealth/ihac049

**Published:** 2022-07-28

**Authors:** J Mwai, J Mutai, L Kaduka, M Abdi, I Ahmed, P Ndemwa, D Nyole, J Omogi

**Affiliations:** Centre for Public Health Research, Kenya Medical Research Institute, Kenya; Centre for Public Health Research, Kenya Medical Research Institute, Kenya; Centre for Public Health Research, Kenya Medical Research Institute, Kenya; Centre for Public Health Research, Kenya Medical Research Institute, Kenya; Centre for Public Health Research, Kenya Medical Research Institute, Kenya; Centre for Public Health Research, Kenya Medical Research Institute, Kenya; Moi University; Jomo Kenyatta University of Agriculture and Technology

**Keywords:** COVID-19, households, Kenya, knowledge, practice, WASH

## Abstract

**Background:**

Coronavirus disease 2019 (COVID-19), declared a global pandemic by the World Health Organization (WHO), is a severe acute respiratory disease. The Kenyan Ministry of Health (MoH) put in place measures that included mandatory face masking, hand and cough hygiene and social and physical distancing to reduce disease transmission and increase prevention efforts. The primary objective of this study was to determine how sociodemographic characteristics affect knowledge and practice of the above measures.

**Methods:**

A cross-sectional study was conducted to assess water, sanitation and hygiene practices for the prevention and control of COVID-19 in Kilifi and Mombasa Counties, Kenya. Data collection was accomplished through a mobile data collection tool. Principal component analysis was used to create a wealth index using data on asset ownership and housing characteristics. Bloom cut-off points of 80–100%, 60–79% and ≤59% were used to determine knowledge and practice.

**Results:**

Of the 612 households, 339 (55.4%) were from Kilifi County and 273 (44.6%) were from Mombasa County. A total of 431 (70.4%) were female and the mean age of the household members was 38.2±14.8 y.

Almost all (99.2%) respondents were aware of COVID-19, with 60% knowing prevention, symptoms and persons at a higher risk of contracting the virus. Females had the highest knowledge of COVID-19 and were likely to practice prevention and control measures, unlike males. Age was significant (p<0.05) with knowledge and practice.

**Conclusions:**

The sociodemographic characteristics of populations play a key role in behavioural aspects as far as prevention and control of COVID-19 are concerned. There is a need for partnerships between the MoH and county governments to put in place a multisectoral community approach to advance feasible behavioural interventions among targeted populations towards combating the spread of COVID-19.

## Introduction

The advent of coronavirus disease 2019 (COVID-19) has presented an unprecedented global threat to human health and economies since the first case was declared in Wuhan, China in December 2019.^1,2^ In March 2020, the World Health Organization (WHO) declared COVID-19 a global pandemic. With no effective treatment or vaccine available then, mitigation and containment measures were adopted across the world to prevent and control the spread of the virus.^3^

While some researchers have referred to COVID-19 as ‘the great equalizer’, early reports from hard-hit areas in the USA suggest that the disease has a disproportionate burden associated with the long-standing social determinants of health, including racial/ethnic and socio-economic disparities.^4^

The Kenyan Ministry of Health (MoH), through the National Emergency Response Committee on Coronavirus (NERCC), put in place several measures to reduce disease transmission and increase prevention efforts. These included mandatory face masking in public, promotion of hand and cough hygiene, dusk to dawn curfew (including initial restriction of movement into and out of hotspot counties), promotion of social and physical distancing, suspension of learning in all educational institutions and promotion of working from home. More recently, the government added home-care management of asymptomatic and mild cases. It also decentralized the COVID-19 response to counties and focused on optimizing the use of a community strategy in managing and controlling the disease.^5^ For this study, we focused on mandatory face masking in public, promotion of hand and cough hygiene and promotion of social and physical distancing.

Knowledge and practice are important factors regarding health prevention and promotion. They involve a range of beliefs and exacerbating factors, identification of symptoms and available methods of treatment and consequences.^6^

There have been genuine concerns about misinformation that have affected public health responses. As expressed by the WHO’s Director-General, Dr. Tedros Adhanom Ghebreyesus, ‘we're not just fighting an epidemic; we're fighting an infodemic’. In low- and middle-income countries (LMICs), households in resource-poor settings may not have access to regular and reliable sources of information about disease aetiology, leaving them ill-equipped to minimize the risk of infection during emerging outbreaks.^7^

Given the importance of sociodemographic characteristics and knowledge and practice in behaviour modification for disease control, it becomes pertinent to assess the association between these important variables. We hypothesized that there was no difference in the sociodemographic characteristics of the households with knowledge and practice of COVID-19 mitigation factors. Therefore we aimed at assessing how sociodemographic characteristics affect knowledge and practice about COVID-19 measures among selected households in Kilifi and Mombasa Counties in Kenya.

## Methods

### Study design and setting

This was a cross-sectional study that collected quantitative data using an interviewer-administered questionnaire that was converted to Open Data Kit (ODK), a mobile data tool. The survey was conducted between 25 November and 3 December 2020 in Kilifi and Mombasa Counties in the coastal region of Kenya. While preparing for the study, the two counties were in lockdown to curtail the movement of people in and out as a way of reducing community transmission of COVID-19,^8^ hence the reason for their inclusion as study sites. According to the Kenya National Census of 2019, the two counties had a combined population of 2 662 120.^9^

According to the Mombasa County Integrated Development Plan (CIDP), the water source in the county is managed by the Mombasa Water and Sewage Company. This supply meets only 65% of the county water demand, with most residents relying on borehole water that contains a high percentage of faecal contamination and is not safe for domestic use. In total, 73.9% of the total population had access to safe water while sanitation coverage in the county stood at 71%.^10^ In contrast, Kilifi County is a general water-stressed county, with a general daily water gap of 80 884 cubic meters per day according to the CIDP, while access to basic sanitation facilities remains a formidable challenge across the county. The county toilet coverage is estimated at 67% and 30% of households have hand washing facilities. A significant proportion of the population in the county has no access to basic sanitation facilities, posing serious public health problems.^11^

### Study population

The study population for the survey included persons >18 y of age who resided in villages that were randomly selected from the wards. A single member of the household was purposively chosen depending on the availability of the head of the household and the socio-economic level of the household. Given that the study was done on a weekday, females were the primary individuals found in the households at that time.

### Sample size determination

The sample size was determined using a formula developed by the Department of Economic and Social Affairs of the United Nations (UN) Secretariat:^12^

\begin{equation*} =\left[ {\frac{{(z^2)*(r)*(1-r)*(f)*(k)}} {{(p)*(\eta)*(e^2) }}} \right] \end{equation*}

where n is the number of households to be selected; z is the confidence level desired (1.96 for 95% level of confidence);

r is an estimate of a key indicator to be measured by the survey (e.g. it is estimated that approximately 36% of Kenyans keep social distance^5^);

f is the sample design effect (deff), assumed to be 2.4 (default value);

k is a multiplier to account for the anticipated rate of non-response (approximately 20% in sensitive surveys in Kenya);

p is the proportion of the total population accounted for by the target population and upon which the parameter, r, is based (approximately 66.6% of the Kenyan population >18 y of age);

ň is the average household size (number of persons per household; approximately 6 according to the Kenya National Bureau of Statistics (KNBS) and

e is the margin of error to be attained (recommended to be set at 10% of r, i.e. e=0.1r). The results are shown in Table 1.

**Table 1.** Sample size determination

County	Households, n	Proportion	Sample households, n
Mombasa	372 292	0.55	339
Kilifi	304 602	0.45	273
Total	676 894	1	612

### Sampling technique

A multistage sampling technique was used to select the villages that were included in the study. The process is described below.

### Selection of the two counties

The two coastal counties were selected given that they were experiencing the highest incidences of COVID-19 apart from Nairobi County at the time of this study.

#### Stage 1: selection of subcounties

Random sampling was employed in selecting three subcounties from a total of six subcounties in Mombasa, while in Kilifi, all seven subcounties were selected.

#### Stage 2: selection of wards

A list of all wards identified from the eight subcounties, totalling 52, were systematically sampled to include only 14, with 3 acting as the nth.

#### Stage 3: village selection

Using probability proportional to size, a total of 28 villages were sampled to be part of the study. The 2019 Kenya population was used in close consultation with the local administration.

A total of 612 households were visited in the two counties. In Mombasa County, the study was conducted in three subcounties: Changamwe, Kisauni and Mvita. In Kilifi County, the study was conducted in all seven subcounties given the low population size. They included Magarini, Kilifi South, Ganze, Rabai, Kilifi North, Kaloleni and Malindi. The survey covered a total of 28 of the 59 villages that were randomly selected in both counties.

### Data collection methods

The survey utilized a quantitative data collection method for the assessment. A validated and standardized questionnaire was converted into an electronic version using an open source mobile data collection tool, Open Data Kit (ODK). The tool was first programmed in xls form, where all constraints and relevance were built before they were uploaded into ODK. This guaranteed a reliable and well-structured questionnaire and made it impossible to skip a question during data collection. The research assistants were trained on how to fill in data in the mobile application and in interview skills. There was a total of six knowledge questions that covered symptoms of COVID-19, the most affected group and sources of information on COVID-19, among others. The respondents were asked whether they wash their hands with soap, wear masks and stay at home. They were also asked the frequency of washing their hands.

Data records were saved after finishing each household interview and could not be edited further, thus protecting the integrity of the data. At the end of the day, the data were uploaded to a secure server and daily summary reports were produced to evaluate daily targets and the completeness of data collection. Upon the collection of data, all the data were converted into Excel (Microsoft, Redmond, WA, USA) and later to Stata version 15 (StataCorp, College Station, TX, USA) for analysis.

### Data analysis

Data were analysed using Stata version 15.0 and descriptive statistics presented in tables and figures. Univariate analysis was done for all variables to compare outcomes of interest. Proportions were used for categorical variables and measures of central tendency and dispersion for continuous variables.

The study also used data on asset ownership and housing characteristic to create a wealth index using principal component analysis. Asset ownership was used because it gives an indication of the longer-term economic status of a household and is less dependent on short-term economic changes compared with other wealth or poverty measures.^13^

Using the Shapiro–Wilk test, knowledge and practice responses were tested for normality before analysis. For the knowledge, attitude and practice (KAP) studies, Bloom cut-off points of 80–100% for high knowledge, 60–79% for moderate knowledge and ≤59% for poor knowledge were used.

Chi-squared and Fisher’s tests were used to determine significant differences in the key outcome variables. Significant levels were set at α=0.05 and logistic regression was done to determine the predicting factors associated with water, sanitation and hygiene practices.

### Ethical approval

We obtained ethical approval from the Kenya Medical Research Institute's (KEMRI) Scientific and Ethical Review Unit (SERU) before conducting the study. A research permit was also obtained from the National Council of Science, Technology and Innovation (NASCOTI). Approval to conduct the studies in the counties was obtained from the Kilifi and Mombasa County health offices.

### Reliability and validity

The study applied a standardized, pretested quantitative questionnaire.^14–17^ Pretesting was done in Likoni subcounty. The sampling procedure used was appropriate to the study and representativeness, given the situation the two counties were in. The study addressed a pertinent issue regarding the role of sociodemographic characteristics in adhering to COVID-19 protocols.

### COVID-19 measures and protocol adherence

Before conducting the study, both the county governments of Kilifi and Mombasa and the research team held a virtual meeting and discussed the best way to avoid COVID-19 infections. Among the requirements were to extend the number of days and work with a small number of research assistants. A total of three vans were used to maintain social distance during traveling. All the research assistants were given masks and were advised to wear them correctly at all times. They were also given hand sanitizers. The team also agreed to rest anyone who developed COVID-19 symptoms. In addition, the research team from KEMRI underwent COVID-19 testing 24 h before traveling to the field.

## Results

### Sociodemographic characteristics

More than half of the households surveyed were from Kilifi County (339 [55.4%]), with women comprising the majority (431 [70.4%]) of individuals interviewed. The mean age of the household heads was 30.8±19.5 y. A majority (56.2%) of the respondents were 20–39 y of age, with 457 (74.7%) of the respondents married at the time of the study. Christianity was the major religion in the two counties and 438 (71.6%) had attended primary school (Table 2).

**Table 2.** Sociodemographic characteristics (N=612)

Variable	Frequency	Percentage
County		
Kilifi	339	55.4
Mombasa	273	44.6
Household head		
Adult female	218	35.6
Adult male	329	53.8
Elderly female	24	3.9
Elderly male	41	6.7
Gender		
Male	181	29.6
Female	431	70.4
Age (years), mean±SD	38.2±14.8	
Age group (years)		
18–27	165	27
28–37	178	29.1
38–47	118	19.2
48–57	67	11
58–67	54	8.8
>67	30	4.9
Marital status		
Married	457	74.6
Single	106	17.3
Widowed	35	5.7
Divorced	14	2.3
Education level		
None	126	20.6
Primary education	438	71.6
Secondary education	48	7.8
Main form of employment		
Self-employed	134	21.9
Farmer	139	22.7
Employed	131	21.4
Unemployed	208	34

### Knowledge of COVID-19

Almost all (99.2%) of the household members were aware of COVID-19, with the most common source of information being radio (79.6%), television (44.2%), health facility/community health volunteers (28.1%) and social media (11.3%). Most of the participants were aware of COVID-19 symptoms, with 74.8% mentioning high fever, dry cough (70.3%), difficulty breathing (57.2%) and headache (51.1%). Others mentioned fatigue (28.5%), sore throat (27.9%) and runny nose (25.8%).

Most households identified the elderly (82.1%), children (57.8%), women (41.4%) and people with human immunodeficiency virus/acquired immunodeficiency syndrome as the groups of people most at risk of acquiring COVID-19.

When asked how they could prevent COVID-19, 94.2% answered wearing a mask, 87.9% said washing hands and 43.5% said social distancing. Others mentioned using sanitizer (32.6%), avoiding crowded places (24.1%), avoiding shaking hands (20%) and staying home (15%), among others.

Regarding actions that individuals or household members should take if they suspected they could be suffering from COVID-19, 89.7% indicated that they would visit the nearest health facility, while 17.4% would isolate themselves, 16% talked of quarantine and 7.9% said they would wear a mask.

A cross-tabulation between sociodemographic characteristics and knowledge showed a strong significance between knowledge and county, sex, marital status, religion and level of education. Generally the knowledge of residents in Kilifi was 2.6 times greater compared with the knowledge of residents in Mombasa. In contrast, females were more likely to have good knowledge compared with males, with the odds of females having good knowledge being 1.6 times higher than males. Further analysis showed that the higher the education one had, the higher the knowledge (Table 2).

In general, 379 (61.9%) of the respondents recorded poor knowledge of COVID-19 symptoms, the persons at risk and ways of prevention.

### The practice of COVID-19 measures

Almost all (96.6%) respondents indicated that they washed their hands, with 54.3% washing their hands three to five times, 37.9% washing their hands one to two times and 7.8% never washing their hands. This was regardless of the period and had no specific time.

In terms of practice, 94.5% reported washing their hands with soap, 86.4% said they wore masks, 68.4% stayed at home and 4.6% talked of reporting to the nearest health facility.

A total of 180 (29.4%) stated that they sometimes stayed at home, 175 (28.6%) always stayed at home and 109 (17.8%) often stayed at home, while 131 (21.4%) stated that they rarely stayed at home and 2.8% never stayed at home. Less than half (46.7%) stated they always put on masks, 15.4% said they often put on masks, while 23.7% 11.9% and 2.3% sometimes, rarely and never put on masks, respectively.

In terms of practice, two-thirds (65.4%) of the respondents had poor practice while 34.6% had moderate.

A statistical significance was recorded between county and practice of COVID-19 measures, with the odds of residents in Kilifi practicing the COVID-19 measures being 1.8 times higher compared with residents in Mombasa. Cross-tabulation between education level and practice showed a strong significance. Age was statistically significant to knowledge, with the odds of respondents <37 y of age having a higher knowledge compared with those who were ≥65 y of age. The odds of practicing COVID-19 measures among the respondents who had a education was 2.2 times higher compared with those who had no formal education (Table 3).

**Table 3.** Association between sociodemographic and water-related indicators

	Source of water			Availability of enough water			Payment of water		
Variables	Non-improved source, n (%)	Improved source, n (%)	Test statistics	Yes, n (%)	No, n (%)	Test statistics	Yes, n (%)	No, n (%)	Test statistics
County									
Kilifi	45 (13.2)	294 (86.7)	χ^2^=2.5 p=0.11	186 (54.8)	153 (45.3)	χ^2^=7.19 p=0.007, p=0.64 (0.45–0.89)	257 (75.8)	82 (24.1)	χ^2^=25.0 p=0.000, p=0.32 (0.20–0.51)
Mombasa	25 (19.2)	248 (90.8)		179 (65.6)	94 (34.4)		248 (90.8)	25 (9.2)	
Gender									
Female	52 (12.1)	379 (87.9)	χ^2^=0.56 p=0.45	259 (60.1)	172 (39.9)	χ^2^=0.12 p=0.72	356 (82.6)	75 (17.4)	χ^2^=0.007 p=0.93
Male	18 (9.9)	163 (90.1)		106 (58.6)	75 (1.4)		149 (82.3)	32 (17.7)	
Marital status									
Married	61 (13.3)	396 (86.6)	Fisher's exact=0.43	266 (58.2)	191 (41.8)	χ^2^=3.5 p=0.319	367 (80.3)	90 (19.7)	Fisher's exact=0.06
Divorced	5 (14.3)	30 (85.7)		25 (71.4)	10 (28.6)		9 (25.7)	26 (74.3)	
Single	4 (3.8)	102 (96.2)		67 (63.2)	39 (36.8)		8 (7.5)	98 (92.4)	
Widowed	0 (0)	14 (100)		7 (50)	7 (50)		0	14 (100)	
Education level									
None	29 (23)	97 (77)	Fisher's exact=0.32	78 (61.9)	48 (38.1)	χ^2^=0.86 p=0.64	95 (75.4)	31 (24.6)	χ^2^=5.6 p=0.06
Primary	39 (8.9)	399 (91.1)		261 (59.5)	177 (40.4)		370 (84.5)	68 (15.5)	
Secondary	2 (4.1)	46 (95.8)		26 (54.2)	22 (45.8)		40 (83.3)	8 (16.7)	
Age group (years)									
18–27	18 (21.2)	67 (78.8)	Fisher's exact=0.09	108 (65.4)	57 (34.5)	χ^2^=7.8 p=0.17	141 (85.5)	24 (14.5)	χ^2^=4.89 p=0.43
28–37	16 (15.4)	88 (84.6)		97 (54.5)	81 (45.5)		151 (84.8)	27 (15.2)	
38–47	15 (22.1)	53 (77.9)		65 (55.1)	53 (44.9)		95 (80.5)	23 (19.5)	
48–57	8 (21.6)	29 (73.4)		42 (62.7)	25 (37.3)		52 (77.6)	15 (23.4)	
58–67	9 (32.1)	19 (67.9)		37 (68.5)	17 (31.5)		44 (81.5)	10 (18.5)	
>67	4 (19)	17 (81)		16 (53.3)	14 (46.7)		22 (73.3)	8 (26.7)	
Income (shilling)									
<2999	24 (19.5)	99 (80.5)	Fisher's exact=0.078	124 (53.5)	108 (46.5)	χ^2^=19.0 p=0.002	196 (84.5)	36 (15.5)	Fisher's exact=0.06
3000–5999	29 (29.9)	68 (70.1)		88 (54.7)	73 (45.3)		122 (75.8)	39 (24.2)	
6000–8999	8 (14.8)	46 (85.2)		57 (65.5)	30 (34.5)		73 (83.9)	14 (16.1)	
9000–11 999	4 (21)	15 (79)		33 (73.3)	12 (26.7)		37 (82.2)	8 (17.8)	
12 000–14 999	1 (5)	19 (95)		27 (84.4)	5 (15.6)		31 (96.9)	1 (3.1)	
>14 999	4 (13.3)	26 (86.7)		36 (65.5)	19 (34.5)		46(83.6)	9(16.4)	

### Wealth quartile

Upon wealth index analysis, the findings showed that 40.3% were in the lowest quartile, 19.8% were in the middle quartile, 19.8% were above average and 20.1% were in the highest quartile. Cross-tabulation between knowledge and practice and the wealth index showed a strong statistical significance.

### Cross-tabulation between knowledge and practice

Cross-tabulation between knowledge and practice showed a strong statistical significance, with the odds of practicing COVID-19 measures being 1.4 times higher among those with good knowledge compared with those with poor knowledge of COVID-19.

## Discussion

A series of interventions have been advanced and implemented in many countries to combat COVID-19, which has resulted in unprecedented consequences, especially in regards to loss of human lives and weakened health systems. Developing countries have had to bear the greatest burden, as their resources and health systems are still developing. In Kenya, the MoH instituted measures in the form of guidelines for prevention and containing the spread of the disease.

The findings of this study reveal that almost all (607 [99.2%]) households were aware of COVID-19. This is similar to a study done in Pakistan in which >90% of medical students were aware of the disease.^18^ In another study in Nepal, 91.6% of the population were aware of all the clinical signs of COVID-19.^19^ In another survey carried out in three African countries, >94% of the population had information about COVID-19.^20^ The high awareness could be attributed to the campaigns that were put in place by MoHs and other partners in different counties.

The finding regarding almost three-quarters of the households being aware of COVID-19 from the radio while 4 of 10 got information from television is similar to a finding in another study in Sierra Leone in which radio was the major source of information on COVID-19.^21,22^ This is in contrast to a study done in Kenya, South Africa and Nigeria in which social media was the most common source of information on COVID-19 while television and radio were second and third, respectively.^20^ However, in a study among medical students, the major source of information was the MoH website.^18^ Our results might be different from others given that in the same period, the MoH primarily used radio and television to communicate information every day to the public informing them of the status of COVID-19 in the country. In addition, although it was beyond this study, there seem to be specific ages that got different information from different sources. However, in this study it was found that television and radio are the key sources of information for the majority of households.

Current findings indicate that most respondents identified the symptoms of COVID-19, including high fever, dry cough and difficulty breathing. This is similar to a study carried out among Egyptians and Nigerians in which most of the respondents were able to identify the symptoms.^16^ A study done in China also showed that >90% of respondents understood the symptoms of COVID-19.^23^ The majority of respondents identified children as high-risk individuals for contracting COVID-19. The figures are higher compared with a study conducted in an informal settlement in Kenya.^14^ This misconception with regards to children's risk of COVID-19 could be a result of the school closures during the same period, hence the thought that children were at risk.

Among the prevention strategies put in place to combat the spread of COVID-19, staying at home was the least mentioned, with females likely to stay at home, unlike males. This could be attributed to the fact that a higher percentage of the workforce is male. In contrast, wearing masks and washing hands were highly mentioned.

The significant associations (p<0.05) observed in this study between education levels and knowledge levels are similar to studies from other KAP studies conducted in Egypt and Nigeria.^16,21,26^ However, a cross-sectional study among pharmacy students in Saudi Arabia found no statistical significance between gender and knowledge levels.^27^

Females were more likely to have higher knowledge of symptoms, persons at risk and COVID-19 preventive measures compared with their male counterparts. The findings are similar to those from a study done among youths in which female respondents were more likely to identify symptoms correctly compared with men.^24^ It is also similar to a study conducted in Bangladesh where females had more correct knowledge about symptoms compared with males.^28^ However, the findings are different from those of a study in India in which, compared with men, women were less likely to know the main symptoms of COVID-19.^29^

According to our findings, females were likely to practice measures put in place to combat COVID-19, in contrast to findings in India and Cameroon in which women were less likely to practice key preventive behaviours compared with men.^29,30^

Study findings revealed that respondents with higher education levels were more knowledgeable about COVID-19 symptoms, persons at risk and preventive measures. The results are similar to other studies^24,25^ in which those with a college education had a higher level of awareness in identifying signs and symptoms compared with those with lower levels of education. In this study, younger respondents had a higher knowledge of COVID-19 compared with older respondents; this could be a result of the higher education level among the respondents, who were ≤37 y of age. This is in contrast to findings from India where age groups <18 y of age (vs 31–35 y) were significantly associated with low knowledge scores.^25^ However, it is important to note that our study did not interview respondents <18 y of age.

This study posted a statistically significant (p<0.05) association concerning practice, geographical, gender and age group with handwashing using soap, staying at home, wearing a mask and reporting cases to the nearest facility. The findings are in tandem with the findings from a study by Alnasser et al.,^31^ where gender and age were statistically significant with practice. Although there is a need for these findings to be interpreted with caution due to the difference in numbers, females were likely to practice the four measures put in place.

## Conclusions

The current study findings reveal that most of the respondents had good knowledge of the COVID-19 pandemic. The study also shows a few unexpected levels of knowledge and practice towards the measures put in place to combat the COVID-19 pandemic. In particular, women seem to be more knowledgeable, likely to have a positive attitude and more likely to practice public health measures. The study also revealed the importance of education level to knowledge of COVID-19 measures. Radio and television remain the biggest source of information about the COVID-19 pandemic. This article demonstrates that the sociodemographic characteristics of the households influence knowledge and practice of COVID-19 mitigation factors.

The need to provide effective targeted health education programs by county governments, specifically the Department of Health, to improve knowledge of COVID-19 is key in addressing sociobehavioural aspects to improve practices of prevention and control of COVID-19.

## Limitations

Our study was done during a period when there were continuous campaigns on COVID-19. During this time, awareness messages were presented across many different platforms. Second, the responses were focussed on the understanding and feelings of the respondents, not checking their ability. Despite the above limitations, we sincerely believe that the findings of this study provide useful insights into the public's perspective on the measures put in place to combat the COVID-19 pandemic.

Additionally, we believe the findings are useful to provide further guidance to the two county governments and health policymakers.

## Supplementary Material

ihac049_Supplemental_File

## Data Availability

All data generated or analyzed during this study are included in this published article (and its supplementary information files).
